# Working Dog Structure: Evaluation and Relationship to Function

**DOI:** 10.3389/fvets.2020.559055

**Published:** 2020-10-20

**Authors:** Chris Zink, Marcia R. Schlehr

**Affiliations:** ^1^Zink Integrative Sports Medicine, Ellicott City, MD, United States; ^2^Independent Researcher, Clinton, MI, United States

**Keywords:** working dog, structure, function, evaluation, assessment, power, coordination, agility

## Abstract

Working dogs help to keep society and individuals safe, secure, and healthy. To perform their varied functions, it is critical to select dogs that are structurally sound and capable of demonstrating power, coordination and agility. Characteristics such as size and substance, head and axial skeletal structure, chest size and conformation, and thoracic and pelvic limb angulation should be evaluated to select the optimal combination of characteristics to suit the tasks to which each dog will be assigned. This review provides guidance on how to evaluate each of these structural components and discusses the contributions of those body parts to a working dog's function.

## Introduction

There are many different types of working dogs – dogs with jobs that help to keep society and individuals safe, secure, and healthy. Some of these dogs work as military, police, search and rescue, detection (bombs, drugs, cash, agricultural products, termites, mold, cancer, etc.) dogs. Others have jobs as dog guides for the blind, hearing assistance dogs, assistance dogs for the disabled, and work in many other capacities to help their human partners. In this review, discussion will be limited to working dogs that help communities, as opposed to assisting individuals. The majority of these dogs work for government institutions, such as the military, police forces, the Transportation Security Administration, Customs and Border Protection, and agriculture defense dogs. These dogs will be referred to using the upper case designation Working Dogs.

Centuries ago, most selective breeding strategies had the goal of producing dogs to assist with specific tasks that helped humans survive and thrive, such as hunting, herding, or capturing vermin. However, in the last 150 years, this tight relationship between structure and function has, in many cases, dissolved as people began to breed specifically for success in the conformation ring, where dogs are judged predominantly on appearance. At the same time, some individuals chose to breed those same breeds strictly for performance competitions, often leading to distinct differences in the structure of performance and conformation lines of the same breed. This has progressed almost to the point where the performance and conformation lines of many breeds have few structural similarities. These differences in structure between different lines/functions within a breed are perhaps most noticeable in the German Shepherd Dog, the Labrador Retriever, the Golden Retriever, and the Border Collie, breeds that are often recruited for use as Working Dogs.

The detailed anatomy of all dogs, including the bones, muscles, tendons, ligaments, innervation, and vasculature is the same (1). However, the ways in which those components vary and are combined in each breed, resulting in their size and shape, constitute structure. Dogs have the greatest morphological diversity of all mammals (2). Further, the cranial and limb morphology of *Canis familiaris* are more variable than in all of the other canid species combined (3, 4). Those differences arise from the functions for which each breed was originally developed, combined with features selected for by the dog fancy throughout the 20th and 21st centuries. How that structure relates to function in Working Dogs is the subject of this review.

The structural requirements of today's Working Dogs are quite varied because these dogs perform such a wide variety of functions. Working Dogs might need the strength to undergo sudden acceleration to their maximal speed or to leap over a tall barrier, but they might also require the physical stamina to stand or walk all day long. Working Dogs might need to search over rubble or in difficult environmental circumstances such as blistering heat or icy, freezing conditions, often wearing heavy body armor. They also might spend the day detecting specific scents amongst thousands of others, requiring intense mental concentration, which can be physically exhausting. Indeed, several differing functions might be required in the same dog. Each working task requires specialized training and activities that place different and often extreme physical demands on the dogs.

## Structure-Function Relationships

Given the many and varied tasks of Working Dogs and the wide variety of structures of different dog breeds, it is important to develop a deeper understanding of structure-function relationships in these dogs. An intensive examination of the peer-reviewed literature on canine structure-function relationships reveals that there are a few specific areas of intense focus, such as examination of the relationships between tibial plateau angle and cranial cruciate ligament insufficiency (5) and of femoral trochlear groove structure and patellar luxation (6).

However, there is a dearth of peer-reviewed publications that discuss overall canine structure and its relationship to function. This problem perhaps arises exactly because dogs vary so greatly in their structure. When planning an experiment that will correlate a canine structural component with its function, where does one start? Many publications use the Labrador Retriever as an example of the “standard dog,” yet the Labrador Retriever's structure (pelvic limb angulation, e.g.,) varies significantly from that of the German Shepherd Dog. Nonetheless, both of these breeds feature prominently as Working Dogs. And within those breeds, individual structural variation can be dramatic.

Our understanding of the biomechanics of movement, of bone leverage and of muscle/tendon/ligament dynamics, while currently incomplete, is constantly evolving with the use of new technology such as body-worn accelerometers, video and animation technology, and 3D printing based on CT data (7, 8). Still, many existing studies using these technologies to examine structure-function relationships use samples of only 3 to 4 dogs. For example, an outstanding study that examined 3D kinematics of just the canine pelvic limb in only 4 breeds of dogs selected for their functional differences (speed vs. strength) produced a huge amount of data (8). It is hard to imagine that the same study could have included a larger number of breeds. A number of studies of racing greyhounds have provided us with new information on the relative importance of thoracic and pelvic limb musculature for speed in this breed (9, 10), and additional studies have compared structure-function relationships in speed vs. strength breeds (11, 12). There are two outstanding studies that examine the relationships between structure (length vs. cross-sectional area) and function of perivertebral and neck musculature in dogs, and their findings are likely applicable to most dog breeds because to the best of our knowledge all dogs have the same muscles (13, 14). By far, the most comprehensive and scientifically based treatise on the subject of structure-function relationships in dogs is the text *Dogs in Motion* by Martin S. Fischer and Karin E Lilje (15). These authors studied kinematics and kinetics using high frequency videography, marker-based movement analysis, force plates, and biplanar X-ray videography in 327 dogs of 32 different breeds, an outstanding feat of biology and engineering. Increasing numbers of publications can be expected in the future as new technology combined with the ability to analyze extremely large data sets improves.

In general, specific breeds and cross-breeds of Working Dogs are selected because of their trainability, instincts, and temperament for the desired tasks, as well as their size and threat potential, providing a visible deterrent to crime. However, there is minimal evidence-based information regarding what specific structures are most desirable for a Working Dog to have superior abilities and a long and healthy career. For example, what pelvic limb angulation (a term for the combination of angles at which the pelvis, femur, tibia/fibula and metatarsal bones naturally meet in the standing dog) is ideal for superior performance as well as health and longevity in a police dog that needs to perform optimally during a full day of both apprehension and detection? What combination of body size, length, height and muscularity is ideal for a military dog that will be transported by helicopter to hot, dry environments to detect explosives for several hours a day?

This review discusses knowledge derived from peer-reviewed publications where that information exists. However, to fill in the significant gaps in our scientific knowledge, this review also depends on the observations of experienced dog breeders and judges regarding structure-function relationships. These are often based on decades of personal experience and observations of the effects of selective breeding over centuries. Many of these structure-function relationships are described in the breed standards, which are written and sometimes also illustrated descriptions of the ideal dog of each breed. The breed standards are established by individuals with decades of experience in the breed who are considered guardians of those breeds. Breed standards are often considered sacrosanct and are not modified without significant consideration and input from individuals experienced with the breed's structure and original functions. Table 1 provides quotes from the breed standards of the German Shepherd Dog, the Belgian Malinois and the Labrador Retriever that describe the breeds' overall structure as they relate to function. This review discusses structure-function relationships in these three breeds because they are the most common breeds used as Working Dogs. However, it is also important to recognize that there are other Working Dog breeds that have been selected for specific functions, such as the Beagles used at airports and shipping ports to detect illegally imported agriculture products or pests, and thus have different size and structure.

In general, there are two ages at which dogs are selected for careers as a Working Dogs. Puppies are often selected for future careers as Working Dogs when they are ready to leave the breeder, usually at around 8 weeks of age. Breeders and judges of canine structure have long observed that structural evaluation of puppies at 8 weeks of age most accurately predicts adult structure. One all-breed judge who has evaluated thousands of dogs as puppies and again as adults has described her procedure for the structural evaluation of puppies (16).

A second age at which Working Dogs are selected is late adolescence or young adulthood. Government agencies frequently purchase young adult, partly trained Working Dogs because at this age, dogs are already demonstrating their working temperament and many of their adult structural features.

## Size and Substance

When evaluating canine structure, it is important to have the dogs positioned in a standardized stance that allows comparison between individuals. In this review we will use the position in which dogs are stood (or *stacked*) to be structurally evaluated in conformation shows. In that stance, the radius and ulna of both thoracic limbs are placed perpendicular to the ground, the metatarsals are placed perpendicular to the ground, and the head is held up with face looking forward (Table 1).

**Table 1:** Structure-Function Components for Breed Standards for Three Working Dog Breeds.

German shepherd dog (effective 1978)	“The first impression of a good German Shepherd Dog is that of a strong, agile, well muscled animal, alert and full of life. It is well-balanced, with harmonious development of the forequarter and hindquarter. The dog is longer than tall, deep-bodied, and presents an outline of smooth curves rather than angles. It looks substantial and not spindly, giving the impression, both at rest and in motion, of muscular fitness and nimbleness without any look of clumsiness or soft living.” “The breed has a distinct personality marked by direct and fearless, but not hostile, expression, self-confidence and a certain aloofness that does not lend itself to immediate and indiscriminate friendships. The dog must be approachable, quietly standing its ground and showing confidence and willingness to meet overtures without itself making them. It is poised, but when the occasion demands, eager and alert; both fit and willing to serve in its capacity as companion, watchdog, blind leader, herding dog, or guardian, whichever the circumstances may demand.” “The ideal dog is a working animal with an incorruptible character combined with body and gait suitable for the arduous work that constitutes its primary purpose.”	
Belgian malinois (effective 1990)	“The Belgian Malinois is a well-balanced, square dog, elegant in appearance with an exceedingly proud carriage of the head and neck. The dog is strong, agile, well-muscled, alert, and full of life. He stands squarely on all fours and viewed from the side, the topline, forelegs, and hind legs closely approximate a square. The whole conformation gives the impression of depth and solidity without bulkiness.”	
Labrador retriever^*^ (effective 1994)	“The Labrador Retriever is a strongly built, medium-sized, short-coupled, dog possessing a sound, athletic, well-balanced conformation that enables it to function as a retrieving gun dog; the substance and soundness to hunt waterfowl or upland game for long hours under difficult conditions; the character and quality to win in the show ring; and the temperament to be a family companion. Physical features and mental characteristics should denote a dog bred to perform as an efficient Retriever of game with a stable temperament suitable for a variety of pursuits beyond the hunting environment. The most distinguishing characteristics of the Labrador Retriever are its short, dense, weather resistant coat … a clean-cut head with broad back skull and moderate stop; powerful jaws… The typical Labrador possesses style and quality without over refinement, and substance without lumber or cloddiness. The Labrador is bred primarily as a working gun dog; structure and soundness are of great importance.”	
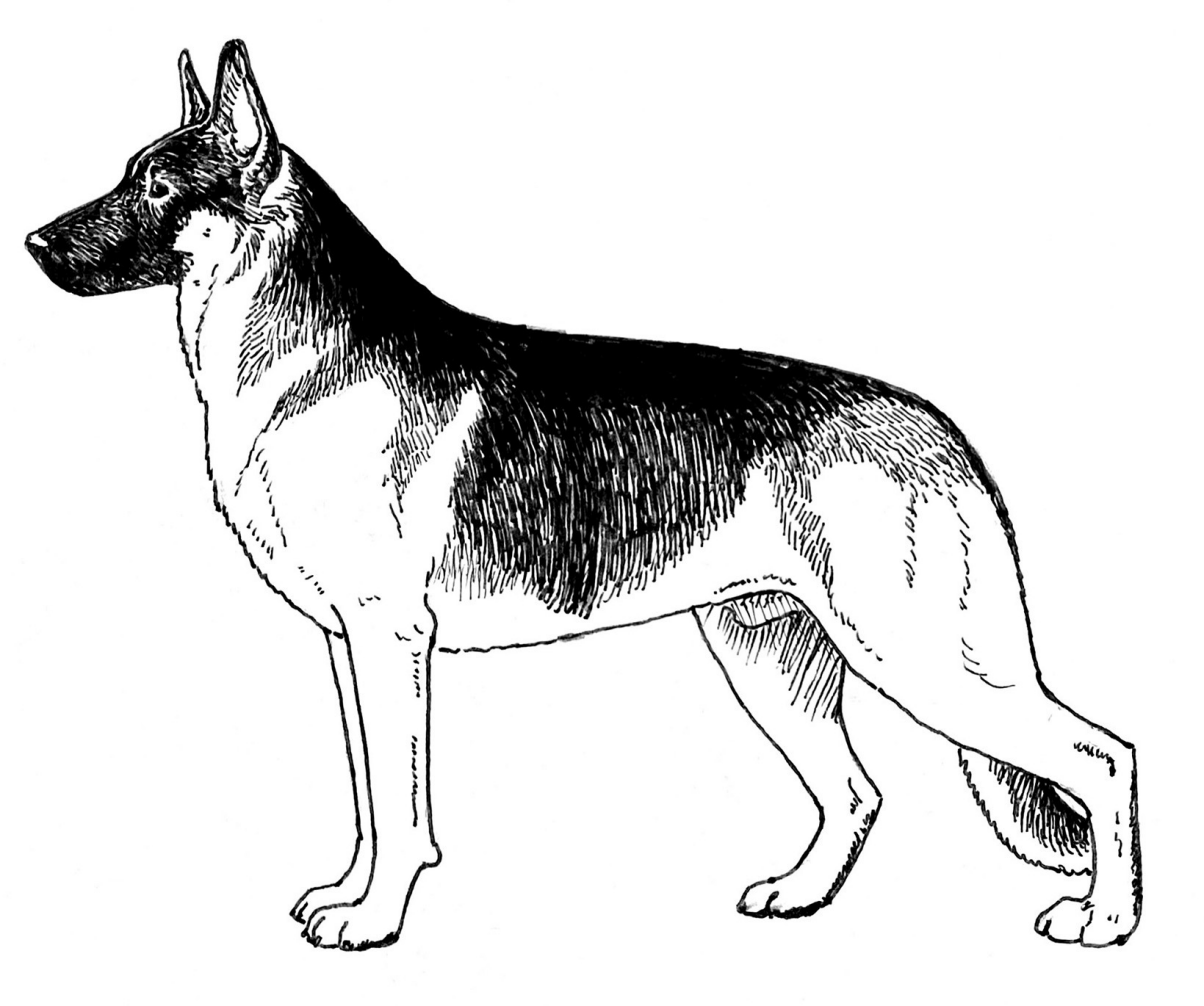	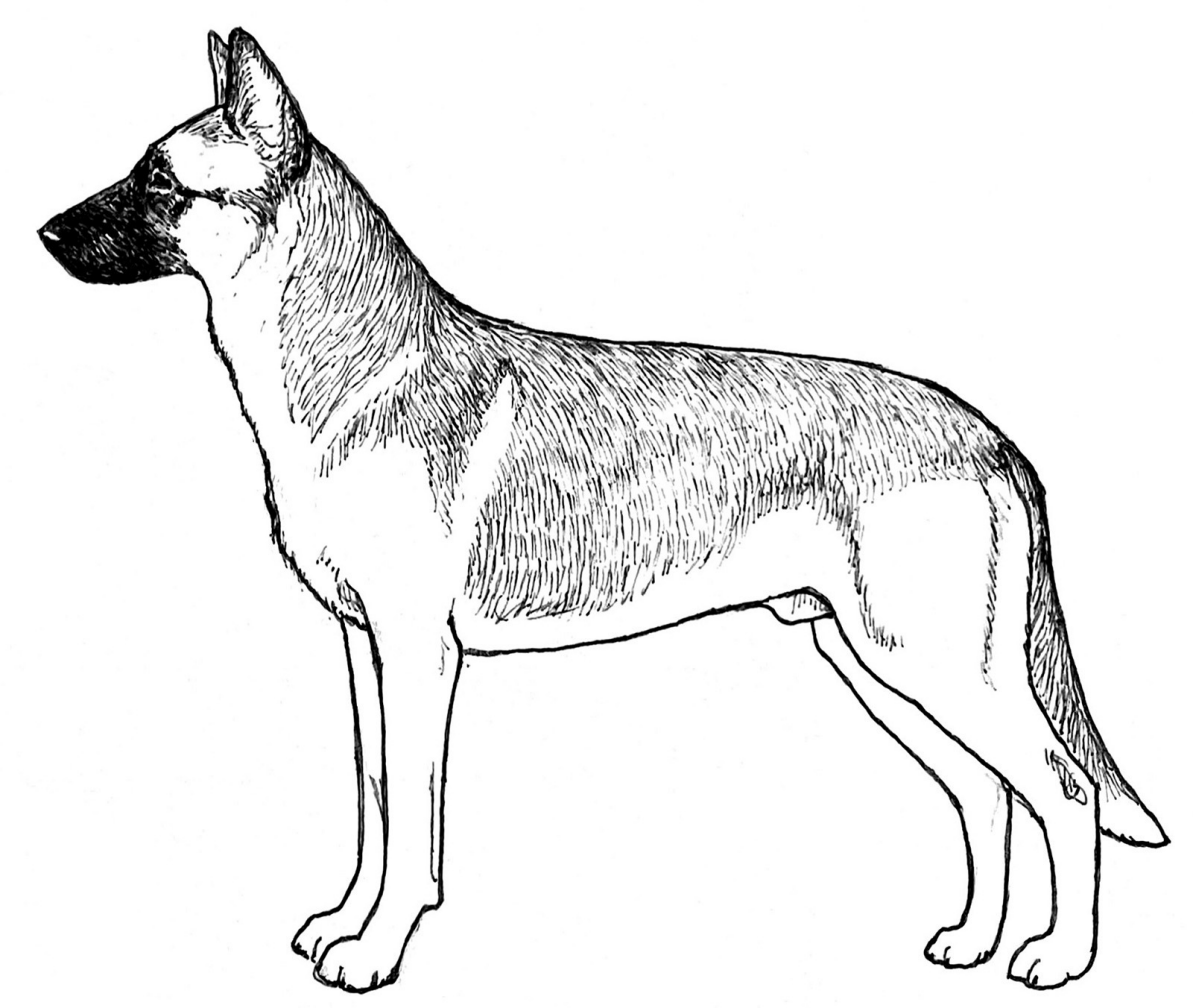	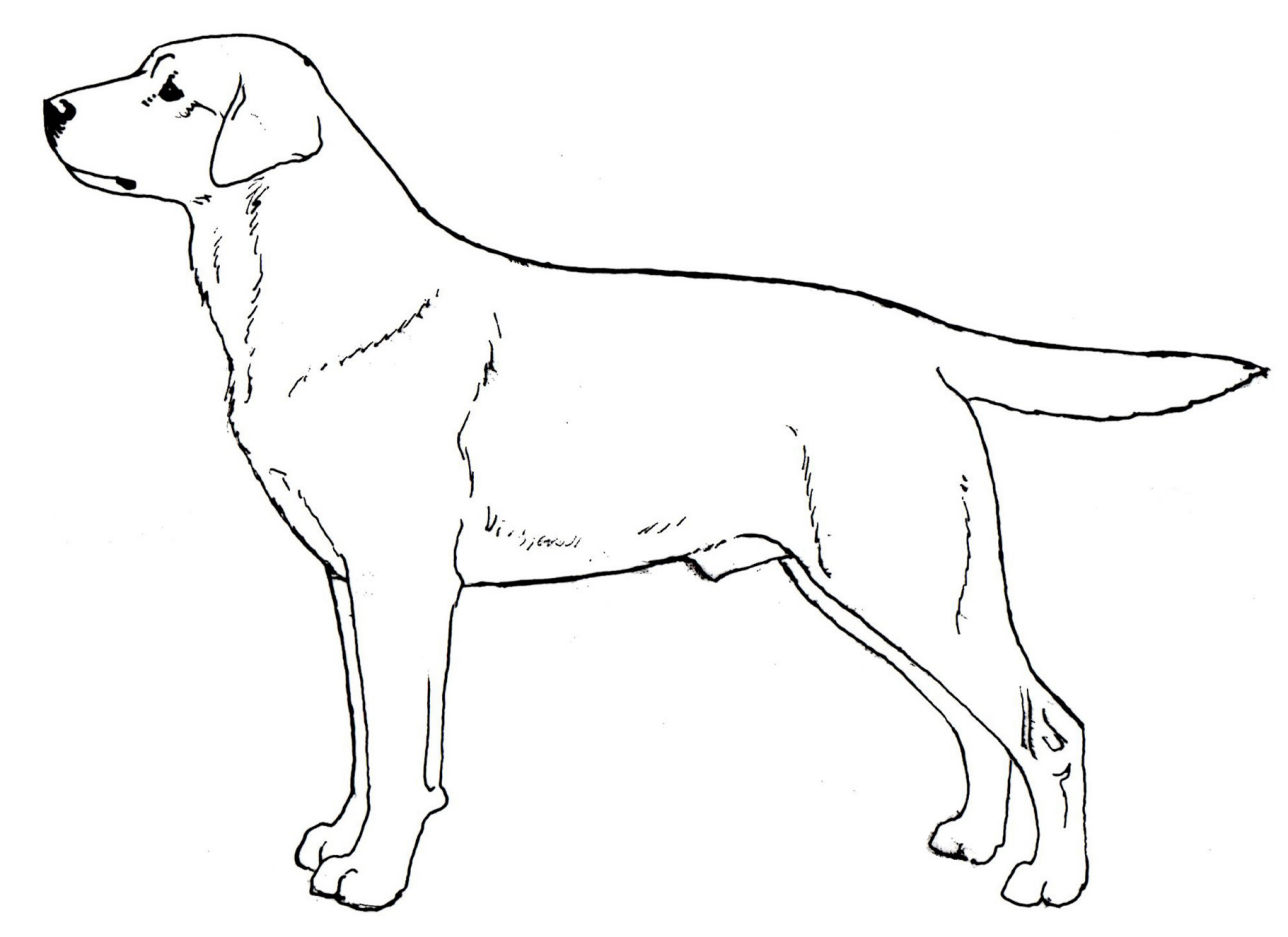
German Shepherd Dog	Belgian Malinois	Labrador Retriever

* Quotes are from the American Kennel Club breed standards. The breed standards of these three breeds can be found at https://images.akc.org/pdf/breeds/standards/GermanShepherdDog.pdf, https://images.akc.org/pdf/breeds/standards/BelgianMalinois.pdf, and https://images.akc.org/pdf/breeds/standards/LabradorRetriever.pdf, respectively.

*Breed standards of the Canadian Kennel Club, The Kennel Club of the United Kingdom, and other registries may differ slightly and can be obtained online*.

It is essential for Working Dogs to have sufficient size and substance to be able to carry out their various functions. For example, during acceleration, the greatest amount of power in the pelvic limb occurs at the coxofemoral joint (10). These forces require not only stable coxofemoral joint conformation, but also optimal development of the muscles that power hip movement. Sufficient size and substance are necessary to produce this power.

**Figure 1:**
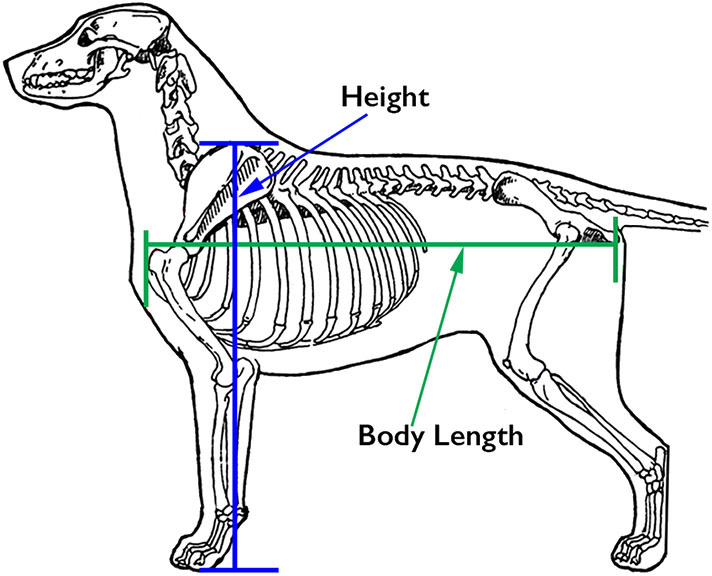
Customary sites for measurement of body length and height. Illustration by M. Schlehr.

When discussing size and substance, the following components are considered: height, generally measured from the ground to the top of the scapula (the *withers*); body length, usually measured from the cranial aspect of the manubrium (the *prosternum*) to the caudal aspect of the ischiatic tuberosity (Figure 1); the relative proportions of the thoracic, lumbar and pelvic components of the body; the dog's weight. Most Working Dogs range in height from 21.5” (53.75 cm) to 26” (65 cm) at the withers, with females generally ~2” (5 cm) shorter than males. The German Shepherd Dog and Labrador Retriever standards both state that the body length should be slightly longer than the height. In contrast, the Belgian Malinois states that these two lengths should be equal. With respect to weight, most Working Dogs lie in the range of 50–80 lb (23–36 kg).

**Figure 2:**
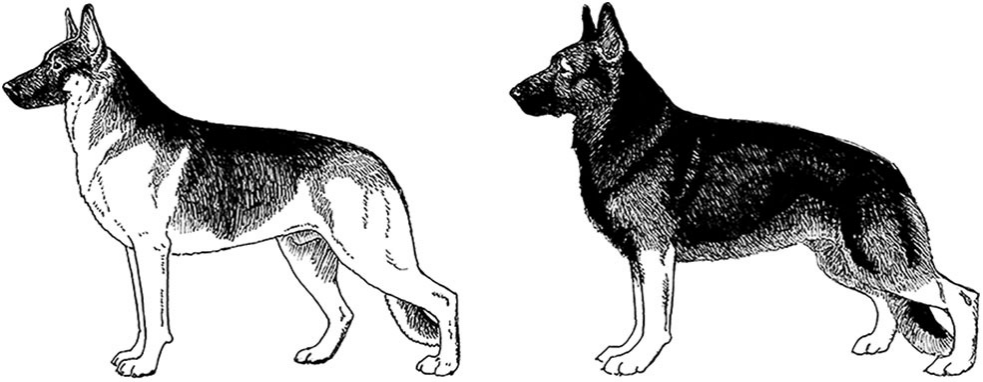
Two German Shepherd Dogs of the same height and overall structure, but with different size and substance. Because of its heavier substance, the dog on the right would be physically less suitable for tasks involving speed and endurance. Illustration by M. Schlehr.

A Working Dog needs to be tall enough to be able to walk at a speed consistent with that of its handler, to run at speeds necessary for chase and apprehension, and have sufficient substance to be able to constitute a substantial threat and stop a fleeing person if necessary. However, moderation in size and substance are also important. All other things being equal, a heavier dog is unlikely to run as fast or have the same endurance as a lighter dog of the same height [Figure 2; (17)]. By the same token, a dog that is lacking in substance might not have the muscular strength to apprehend a large man or to carry the weight of equipment and/or an armored vest throughout an active day. Most individuals of the German Shepherd Dog, Belgian Malinois and Labrador Retriever breeds have balanced combinations of size and substance sufficient to carry out their functions as Working Dogs, although particularly heavy-set or small, weedy individuals should be avoided during the selection process.

Working Dogs should have a large chest for sufficient lung capacity, but the chest should not be so wide as to interfere with gait, as happens with bulldogs, for example (8). Therefore, it is desirable to have the rib cage occupying a large part of the body length, certainly more than half and probably closer to two-thirds of the distance between the manubrium and the ischiatic tuberosity, and to have sufficient depth of chest as well. The lumbar component of the spine provides for a great deal of spinal flexibility, both dorsoventral and lateral, but should also be well-muscled to prevent hyperflexion, particularly during sudden or unexpected movements as can happen during apprehension. Paraspinal and abdominal muscles should be firm to the touch on the standing dog.

### Neck and Topline

The *topline* consists of the upper profile of the dog from the top of the head to the base of the tail. The neck and topline reflect the positioning of the axial skeleton, which supports the ribcage and pelvis and forms a structure for the attachment of the thoracic and pelvic limbs. The axial skeleton is wrapped in the core musculature, which is critical for all components of movement (14). Most students of canine structure and function believe that the neck should be of medium length (18). A long, thin neck lacks the strength to carry heavy objects or support and stabilize the dog during apprehension. A short neck will prevent the full use of the head as a counterbalance and can inhibit thoracic limb movement. The neck should merge with the shoulders gradually; an abrupt junction between neck and shoulders is believed by those who study canine structure to indicate less than ideal shoulder structure.

The *backline* is the part of the topline from the withers caudally. This should be strong and level in Labrador Retrievers and Belgian Malinois and slightly sloping from cranial to caudal in German Shepherd Dogs from working lines; this slope can be very extreme in German Shepherd dogs from conformation lines. The effects of this extremely sloped topline on the dog's strength and mobility have not been objectively studied. A topline that sags in the middle (lordosis) usually indicates weak core (paraspinal and abdominal) musculature but can also be indicative of abnormal vertebral structure. A kyphotic (roached) back is often an indicator of pain, although many German Shepherd Dogs that are bred for conformation have this structure. The effects of this altered axial skeletal conformation on function also have not been objectively studied. Note that all dogs have a normal, small dip in their topline at T11. The dorsal spinous processes of the cervical vertebrae and first 10 thoracic vertebrae are pointed dorsocaudally, while those of the vertebrae caudal to T11 are pointed dorsocranially, and the dorsal spinous process of T11 (the anticlinal vertebra) is very short to accommodate this change in direction of the spinous processes, creating a slight depression.

## Thoracic Limb Structure

There are few peer-reviewed publications on the relationships between canine thoracic or pelvic limb structure and function. As a result, we are dependent on the observations of individuals who have spent decades observing dogs and correlating structure with efficiency of movement and performance longevity. Three of these individuals have published their observations in excellently illustrated books (19–21). The discussions of structure presented here represent an amalgam of their observations, with the results of peer-reviewed publications inserted where such exist.

### Thoracic Limb Angulation – Side View

When evaluating structure of the thoracic and pelvic limbs, it is important to be able to observe and/or palpate the bones as they exist under the skin and soft tissues. The term *thoracic limb angulation* is used by those who study canine structure to describe the angle at which the scapula lies off of vertical and the angles at which the scapula, humerus, and radius and ulna meet at the shoulder and elbow joints, respectively, when the dog is standing in the standard, stacked position. Together, these angles help to determine the ability of the thoracic limb to carry out all of its functions in both moving and stabilizing the body. In most breeds the thoracic limbs bear approximately 60% of the dog's weight when standing, walking and trotting, and they bear the entire weight of the dog in addition to the effects of gravity when the dog is landing from a jump and when the thoracic limbs are bearing the dog's weight during the gallop. The thoracic limbs also function in providing lift at the initiation of jumping. Although in the past, the thoracic limbs were thought to function more for stabilization than forward drive in the moving dog, recent studies suggest that they also play an important role in providing propulsion for forward motion (12).

When the dog is moving forward, abundant thoracic limb angulation, along with optimal musculature, allows the limb to unfold and reach well forward ahead of the dog, to pull the dog's body forward while supporting its weight. Correct angulation and strength also permit the thoracic limbs to extend far caudally, allowing for a long stride length, and to provide lift before beginning the swing phase of the stride in which the dog again reaches forward. Since taking short or long strides require approximately the same amount of energy, it is an advantage to take fewer strides when moving from A to B. At the same time, taking excessively long strides can reduce stability, since stability decreases the further the paw is from the center of gravity. As is so often the case, a balance between stability and forward motion is required.

Thoracic limb angulation is most readily evaluated by assessing two specific features: the angle at which the scapula lies off of vertical and the relative length of humerus, which also determines the angles at the shoulder and elbow joints (19, 20). Scapular angle and length of humerus appear to have different inheritance. Together, they significantly affect the efficiency of thoracic limb function.

#### Angle of Scapula

Movement of the shoulder blade along the rib cage makes up at least 65% of the stride length in dogs (15). A lack of bony attachment of the scapula to the axial skeleton provides for increased range of motion of the thoracic limb, and the angle at which the scapula lies against the ribs is an important factor in allowing extension of the shoulder joint and thus free movement of the entire thoracic limb. To evaluate thoracic limb angulation, the dog should be positioned in the stacked position with the radius and ulna perpendicular to the ground, the metatarsals perpendicular to the ground, and the head held up and muzzle approximately parallel to the ground. This standardized position allows angulation of the thoracic limb to be evaluated in a consistent manner and permits dog-to-dog comparisons.

**Figure 3:**
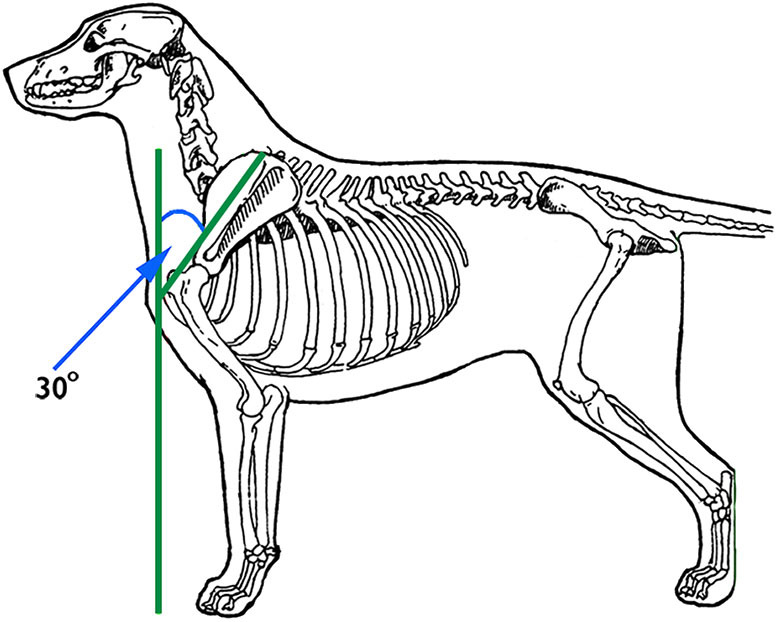
The angle of the scapula is determined by imagining a line perpendicular to the ground that passes through the cranial-most aspect of the greater tubercle of the humerus, then imagining another line that starts at the cranial aspect of the greater tubercle of the humerus and extends to the dorsal-most aspect of the dorsal rim of the scapula. Illustration by M. Schlehr.

The angle at which the scapula lies off of vertical is also referred to as *shoulder layback*. It is determined by imagining a line perpendicular to the ground that passes through the cranial-most aspect of the greater tubercle of the humerus, then imagining another line that starts at the cranial aspect of the greater tubercle of the humerus and extends to the dorsal-most aspect of the dorsal rim of the scapula (Figure 3). This angle at which these two lines meet ideally is 30° based on cineradiographic imaging studies (20). Many books and breed standards describe the correct angle of scapula as 45°, but without objective substantiation (22). This angle can be assessed, with some difficulty, using a goniometer with one arm laid along a straight edge set perpendicular to the ground and abutting the cranial aspect of the greater tubercle of the humerus, and the other arm extending from the cranial aspect of the greater tubercle of the humerus to the dorsal-most part of the dorsal rim of the scapula. More often, the scapular angle is assessed subjectively by placing the thumb and index finger of one hand on the dorsal-most aspect of the two scapulae and comparing how far caudally they are positioned relative to other individuals of the same or other breeds. The more caudally the dorsal-most aspect of the rim of the scapula is positioned, the greater the angle of the scapula. Sufficient angle of scapula is desirable because it allows greater shoulder joint extension and thus more forward reach of the thoracic limb.

Dogs with greater angle of scapula tend to have more developed shoulder muscles, particularly the supraspinatus, infraspinatus and triceps muscles. This might be because these three muscles support the shoulder joint in its angled state in the standing dog. If the scapula lies in a more vertical position, the bones might play a larger role in support. Dogs with a greater angle of scapula are thought to experience less concussion on the shoulder joint particularly when landing with the limb in extension, such as when landing from a jump or when the dog is in a gallop. This is because the well-angled shoulder with greater shoulder muscle strength and greater length of the muscle/tendon units can better flex to absorb the shock of landing and elongate to withstand eccentric contraction of the supraspinatus and biceps muscles as the dog's body falls forward. Resistance to injury from eccentric contraction of these muscles is important given that tendinopathies of these two muscles are amongst the most common injuries in active dogs (23).

#### Length of Humerus

**Figure 4:**
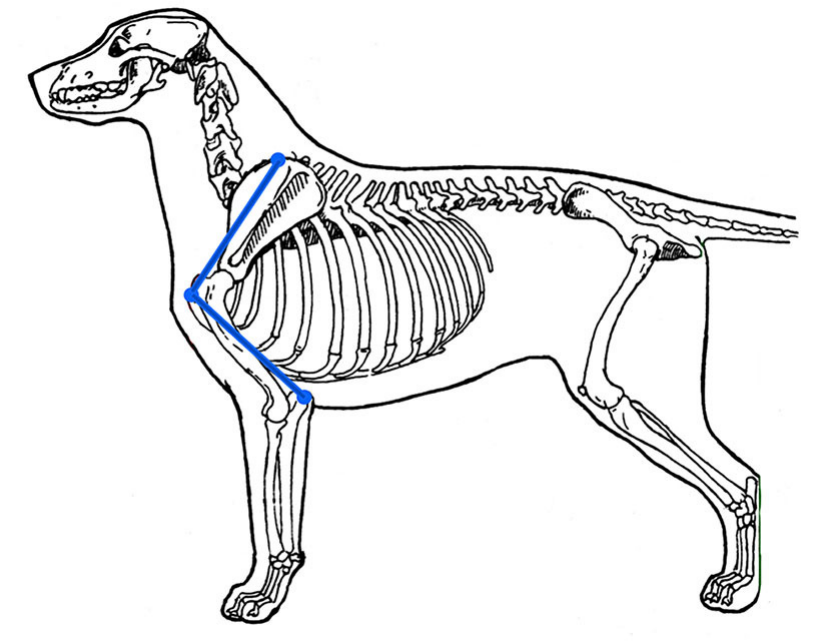
For ideal humeral length, a line drawn from the olecranon process to the cranial aspect of the greater tubercle of the humerus should be the same length as one drawn from the cranial aspect of the greater tubercle of the humerus to the dorsal-most aspect of the scapula. Illustration by M. Schlehr.

A second structural variable of the canine thoracic limb is the length of the humerus, which largely determines the angles of the shoulder and elbow joints. Ideally, the humerus should be long enough to place the dog's radius and ulna in a caudal position, where it can help to support the weight of the chest, when the dog is standing with the radius and ulna perpendicular to the ground. It has been observed by those who study canine structure, that in a dog with an optimal length humerus, a line from the dorsal rim of the scapula to the cranial aspect of the greater tubercle of the humerus is equal in length to a line drawn from the cranial aspect of the greater tubercle of the humerus to the olecranon process in the standing dog (Figure 4). These lengths are easily measured using a flexible tape measure.

**Figure 5:**
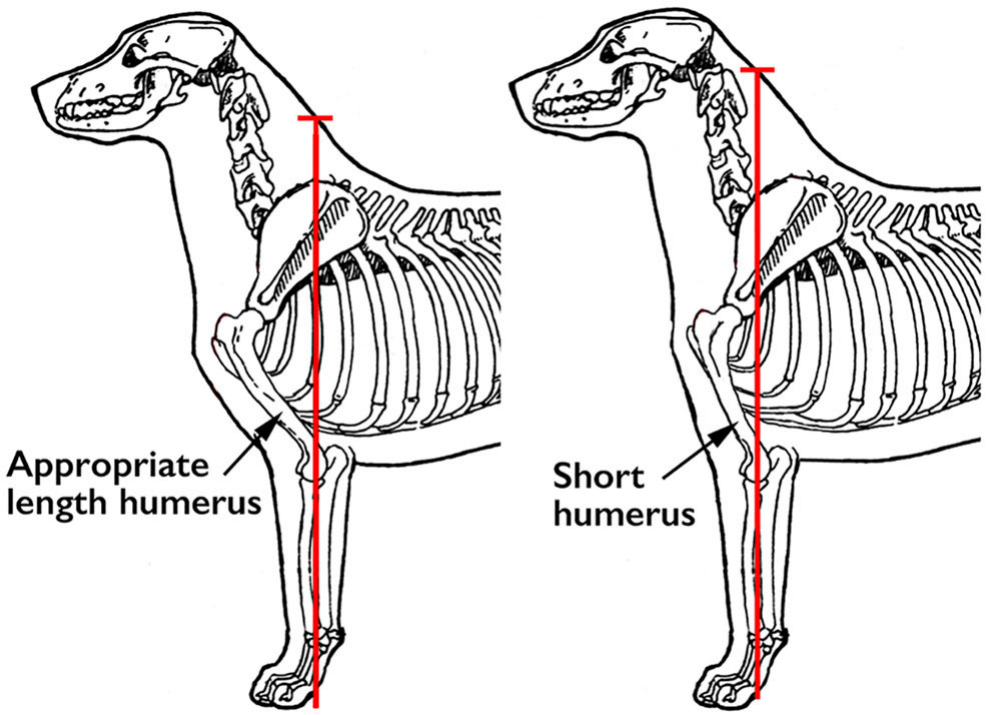
Ideally, a line drawn perpendicular to the ground through the center of the radius and ulna should intersect with the topline at the junction of the neck and back (the withers). Illustration by M. Schlehr.

Another way that dog breeders and judges evaluate humeral length is to imagine a line drawn perpendicular to the ground through the center of the radius and ulna on a stacked dog. This line should intersect with the dog's topline at the junction of the neck and the back (the *withers*). In a dog with a short humerus, the distal thoracic limb is positioned more cranially, resulting in a line that intersects the topline further cranially along the neck (Figure 5).

Dogs with a short humerus have less acute angles at the shoulder and elbow. This might be the reason why it has been observed that these dogs tend to have less well-developed thoracic limb musculature, since they do not have to support these joints in a more angled position. Logically, this would produce more concussion on the bones of these two joints during movement, and more stress on the extensor muscles for these joints during eccentric contraction. To the extent that scapular angle and/or humeral length deviate from ideal, thoracic limb function will be compromised.

Limb angulation does not remain static throughout a dog's life; it changes in response to injury and level of fitness. Dogs with injuries to the thoracic or pelvic limb often experience disuse muscle atrophy. As a result, they frequently stand with less acute angles of the joints, letting the bones stacked one above the other take over more of the function of supporting the limbs. In addition, since it takes muscular effort to support a well-angulated limb, if a dog is not optimally fit, it will have less than optimal thoracic and/or pelvic limb angulation. Measuring the degree of limb angulation when the dog is standing naturally is one way to monitor progress during rehabilitation.

### Thoracic Limb - Front View

**Figure 6:**
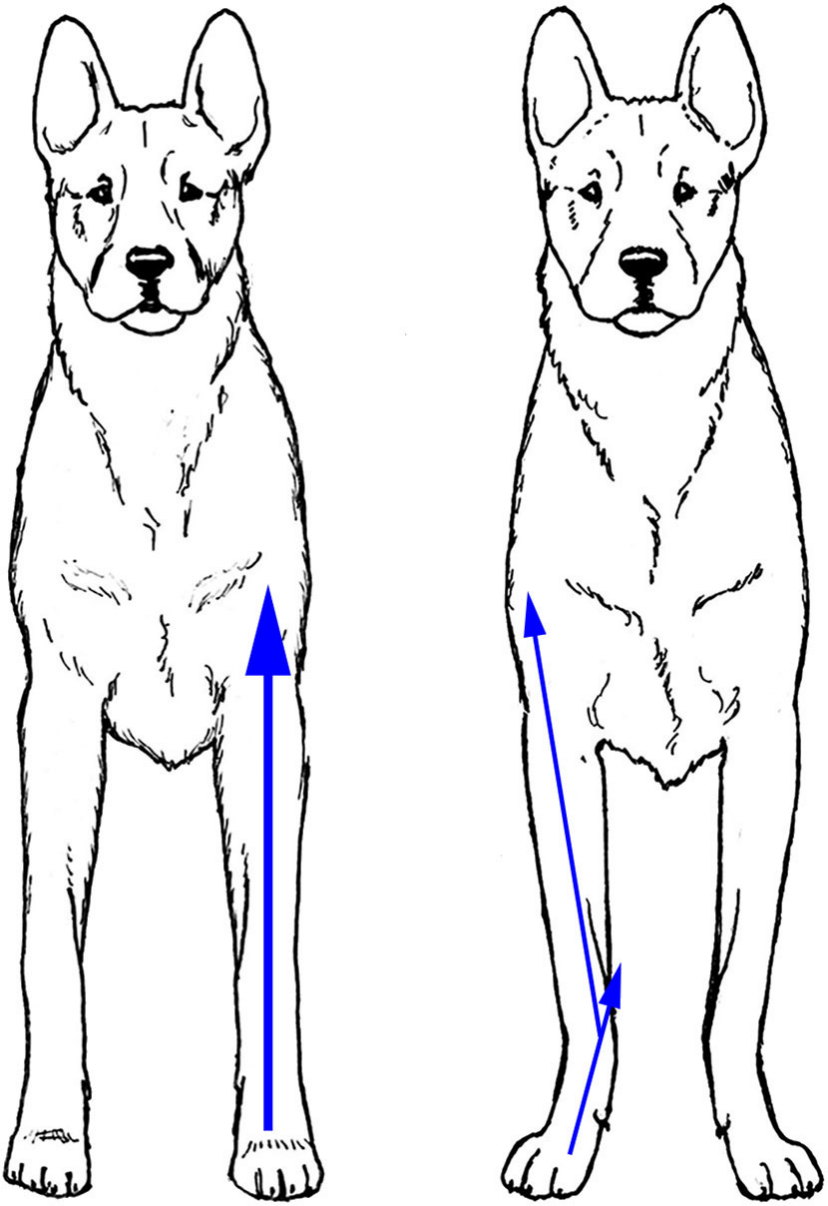
When viewed from the front, the thoracic limbs should form a straight line perpendicular to the ground **(Left)**. Angled limbs **(Right)** dissipate the power output during movement (arrows). Illustration by M. Schlehr.

For the thoracic limbs to function optimally in movement, they have to be able to grip a stable substrate (usually the ground) and then use muscular strength to transfer power along the length of the limb in a sagittal plane to propel the body (9). The most effective way to transfer power is in a straight line. As a result, the standing dog's thoracic limbs, when viewed from the front, should form a straight line perpendicular to the ground from the foot to the body, with minimal bend at carpus or elbow, as demonstrated by the dog on the left in Figure 6. When the thoracic limbs are not straight when viewed from the front, as in the dog on the right in Figure 6, which demonstrates bilateral carpal valgus deformities, the same amount of muscular effort results in dissipation of the power, reducing the effect of the power output on movement. In addition, this can increase stress on the lateral and medical collateral ligaments and tendons that support the joint.

**Figure 7:**
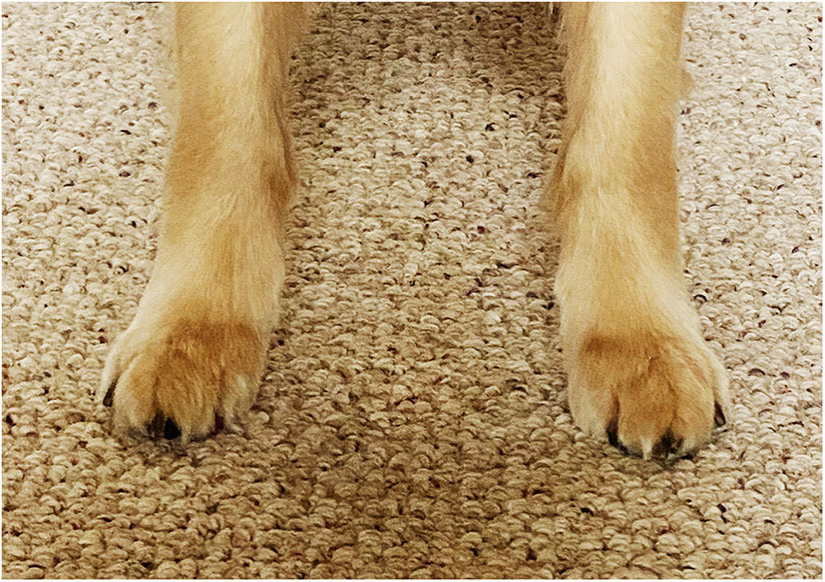
When a dog is standing relaxed, it is normal for the thoracic limbs to be externally rotated, resulting in the feet being positioned with the toes pointing away from each other. This provides stability in the standing position.

In the relaxed standing dog, it is normal for the thoracic limbs to be slightly externally rotated resulting in the feet being positioned with the toes pointing slightly laterally (Figure 7). It is believed that this rotation provides stability for the standing dog, just as a lateral position of the feet do in the standing human, and should not be confused with the valgus deformity seen in the right panel of Figure 6. When the dog in Figure 7 gaits, the thoracic limbs rotate on their axes, thanks to the rotational movements of the radius and ulna, and the feet strike the ground with the toes pointing cranially and no bend at the carpus, providing the most efficient grip on the ground and transfer of power to the body. In contrast, when a dog with valgus deformity gaits, the foot remains externally rotated and the carpal deformity persists.

### Feet and Dew Claws

**Figure 8:**
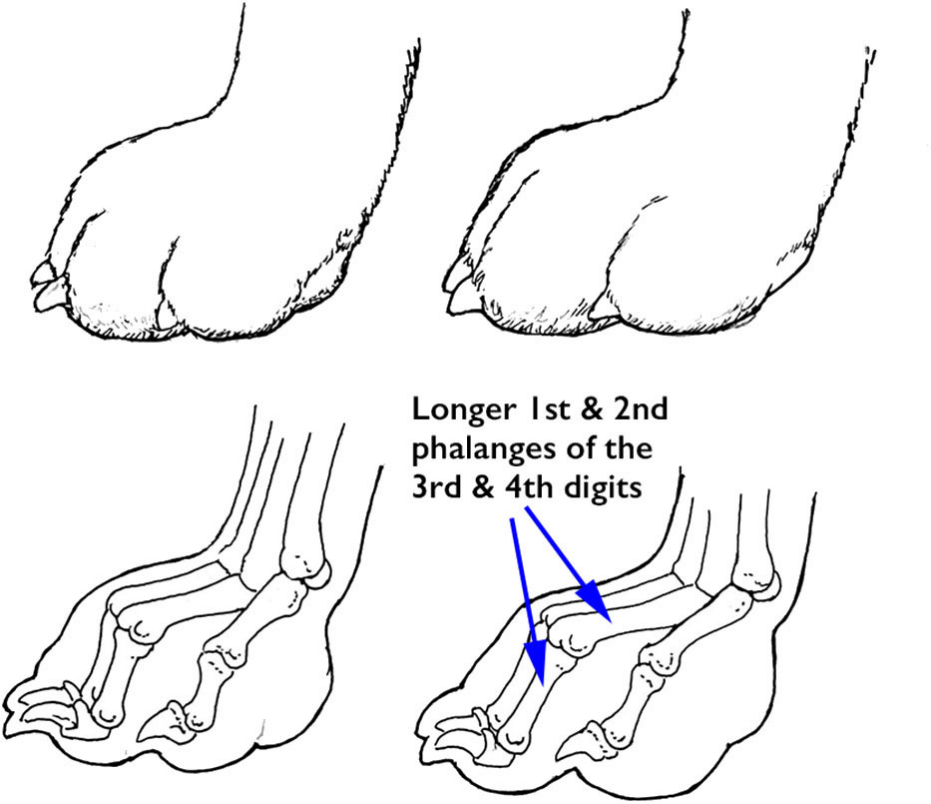
Cat **(Left)** and hare **(Right)** feet, demonstrating the longer 1st and 2nd phalanges of the 3rd and 4th digits in the hare foot (arrows). Illustration by M. Schlehr.

The shape of dogs' feet varies depending on the dog's original function. Dogs that were bred to move over rocky or uneven ground, tend to have compact feet (termed *cat feet*). Cat feet have toes that are all of equal length, forming a half-circle around the central pad (Figure 8). These feet are often considered analogous to the knobby tires of an ATV, that are designed for improved grip on uneven surfaces. Indeed, many breeds with cat feet were bred to be agile moving in all directions or over rough ground. A typical instance is the Afghan hound, which was bred to hunt agile prey over rocky ground.

In contrast, dogs that were bred to run fast in relatively straight lines, such as the Greyhound, tend to have a more elongated foot shape (termed *hare feet*). In these feet, the first and second phalanges of the third and fourth digits are longer than those of the second and the fifth digits, so those toes are longer. An elongated foot is thought to provide an advantage when running straight ahead and is somewhat analogous to the slick tires of a race car, which provide additional grip for forward motion.

**Figure 9:**
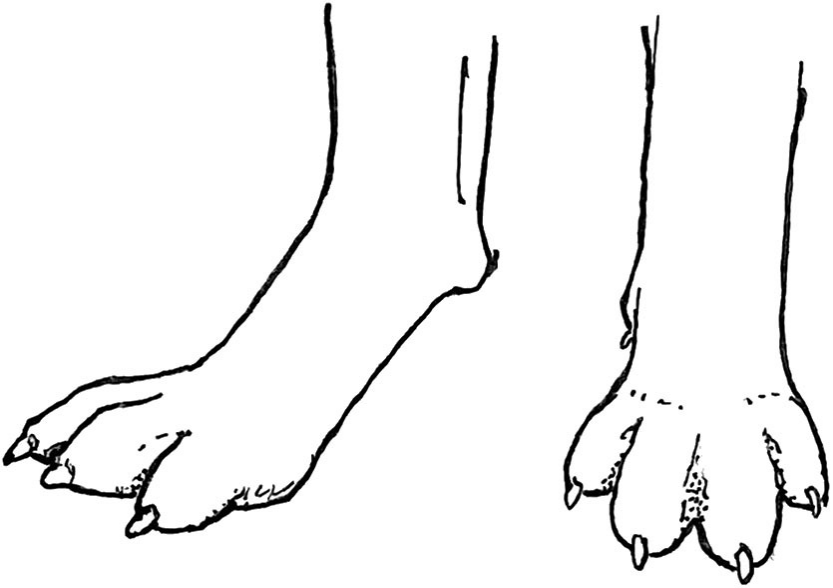
Many German Shepherd Dogs bred for conformation have splayed toes, which might be a reflection of the generalized increased laxity of ligaments and tendons in this breed. Illustration by M. Schlehr.

The superficial digital flexor tendon inserts on the distal second phalanx of each toe, so the dog's toes are spring-like, allowing for improved impact absorption. Repetitive strain to the superficial digital flexor tendon of one or more toes can cause permanent lengthening of these tendons. This results in an increase in the angle of extension at the carpus, and flattening of the phalanges, reducing the ability of the carpus and feet to absorb impact. The breed standards for all three breeds under consideration in this review call for compact feet, and the Belgian Malinois standard specifically states, “The feet are round (cat footed) and well-padded with the toes curved close together” while the rear feet “may be slightly elongated.” Practically speaking, however, most German Shepherd Dogs today tend to have excessive angle of extension at the carpus, and elongated, rather than round, feet. In those German Shepherd Dogs that have more thoracic and pelvic limb angulation, the toes are often, but not always, splayed (Figure 9), although an individual of any breed can have splayed feet. This is thought to reduce the ability of the toes to work as a unit and to increase the risk of toe injuries, since a single toe can be more easily separated from the others, resulting in medial or lateral collateral ligament sprain.

All dogs are born with a first digit on the thoracic limb, also known as the dew claw. Many dogs have their front dew claws removed as 3-day-old puppies because their breeders wish to reduce the risk of dew claw injuries. Breeders of conformation dogs also believe that the absence of a dew claw makes the legs appear straighter when viewed from the front. None of the breed standards of the German Shepherd Dog, the Belgian Malinois or the Labrador Retriever require the dew claws to be removed, and in fact less than a handful of the ~200 breed standards do.

**Figure 10:**
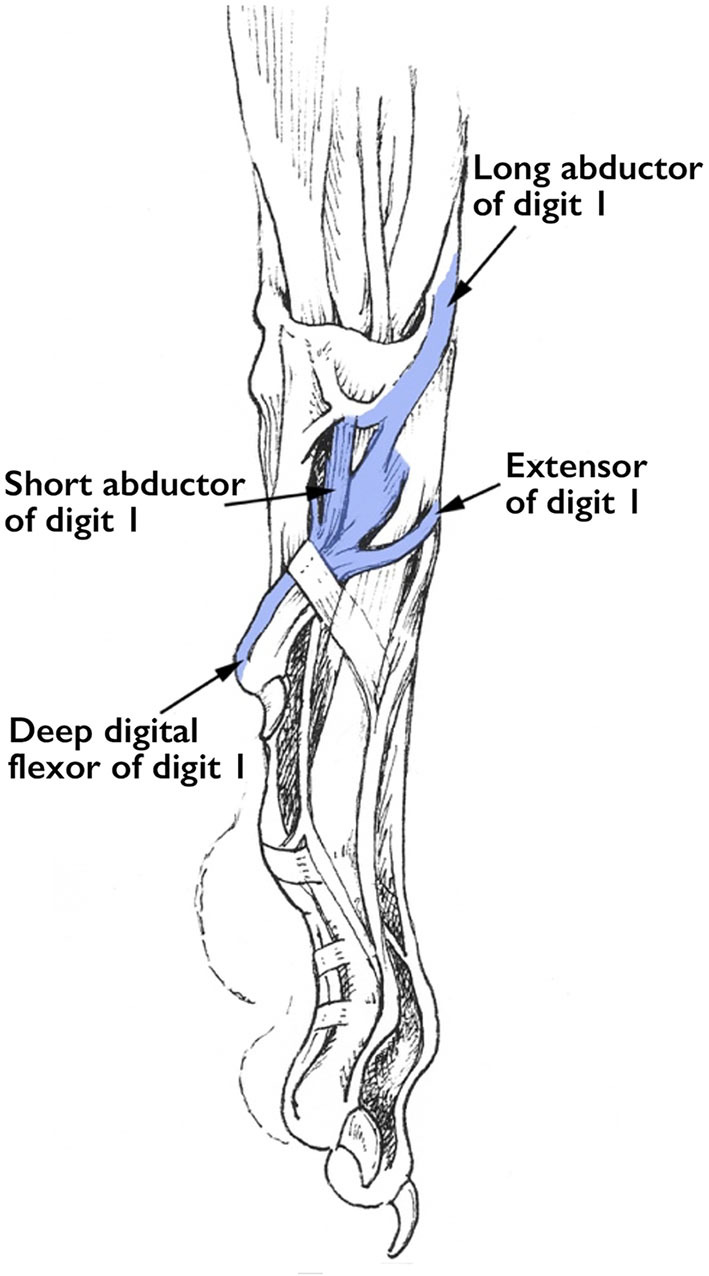
Tendons that attach to the thoracic limb dew claw. Illustration by M. Schlehr (from Miller and Evan's Guide to the Anatomy of the Dog).

Examination of the muscles and tendons attached to the front dewclaws confirm that these digits are functional (1). Four tendons that connect the dewclaw to muscles of the distal thoracic limb (Figure 10) demonstrate that this digit does have the ability to move individually. To the best of our knowledge, all wild carnivores with the exception of African wild dogs have front dewclaws, providing evolutionary proof that they are functional digits.

**Figure 11:**
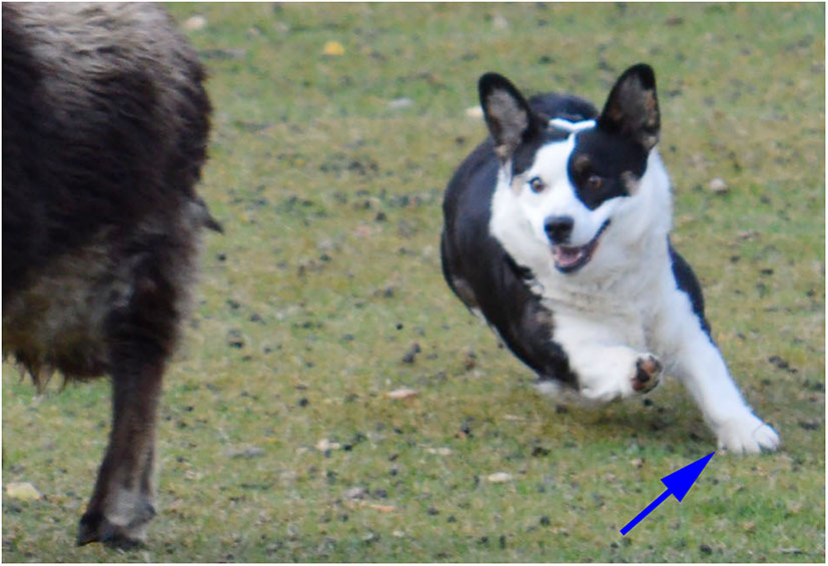
A Corgi herding a sheep demonstrating the use of its left thoracic limb dew claw (arrow) in turning.

The front dew claws appear to be non-functional when the dog is in a standing position because they are not in contact with the ground. However, when dogs are cantering, galloping or jumping and thus bearing the majority of their weight on the thoracic limbs, the dew claw does contact the ground (Figure 11). It is then available to dig into the ground to help stabilize the thoracic limb and reduce torque to the carpus and proximal limb when the dog is turning. An unexpected function of dew claws is to help dogs climb out on ice when the dog accidentally slips through the ice of a pond (or intentionally goes swimming in freezing water). In their position on the medial aspect of the thoracic limbs, they can act as little ice picks to help the dog grip the ice and lift itself out of the water. As a result, many individuals who train performance and working dogs recommend that dew claws not be amputated.

Dew claws on the pelvic limb are almost always vestigial and lack the tendinous attachments of the thoracic limb dew claws. They generally are removed within a few days of birth, except in those breeds such as the Beauceron, Briard, Great Pyrenees, Icelandic Sheepdog, and some others for which the breed standard specifies the presence of rear dew claws.

## Pelvic Limb Structure

### Pelvic Limb Angulation – Side View

**Figure 12:**
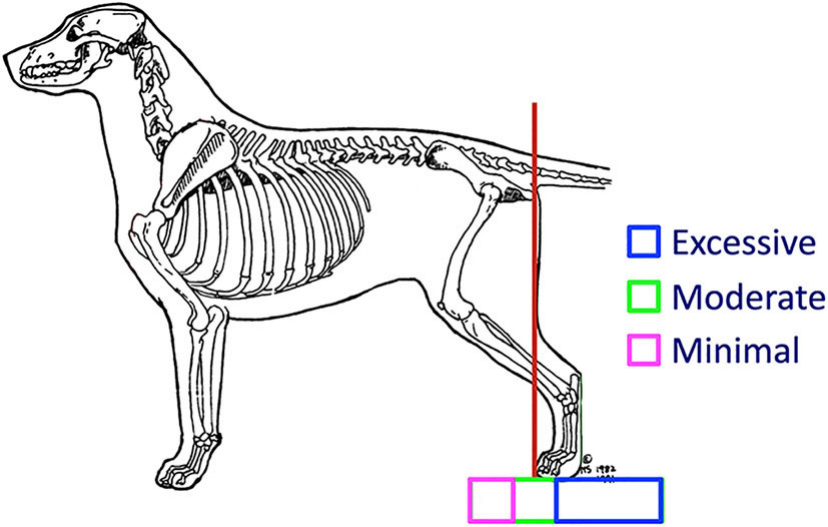
A dog has ideal (moderate) pelvic limb angulation when a line drawn perpendicular to the ground touching the caudal aspect of the ischiatic tuberosity meets the ground at the cranial aspect of the toes (red line). Illustration by M. Schlehr.

Pelvic limb angulation, the angles at which the pelvis and long bones meet one another when the dog is standing, varies widely between different breeds and also between individuals within those breeds. Those who study and evaluate canine structure often refer to this as *rear angulation* by (19–21). As with other structural evaluations, pelvic limb angulation is best assessed by having the dog stand in the stacked position, with the metatarsals oriented perpendicular to the ground. A rule of thumb used by those who study canine structure to evaluate rear angulation is to draw an imaginary line perpendicular to the ground along the caudal aspect of the ischiatic tuberosity (Figure 12). Ideally that line should pass through the cranial aspect of the toes, or within a half of the dog's foot length cranial or caudal to that point.

There are advantages and disadvantages to having either minimal or excessive pelvic limb angulation. Dogs with abundant pelvic limb angulation are able to unfold their limbs to reach farther forward with each stride, powering the body further forward as they extend their pelvic limbs far caudally before lifting the foot for the swing phase of the stride. Excessive pelvic limb angulation, however, is often associated with instability. Since the majority of the pelvic limb musculature is in the proximal part of the limb, there is minimal musculature to stabilize the distal pelvic limb, particularly the tarsus, against lateral or rotational movement. In addition, as with the thoracic limb, stability decreases the further the foot is from a position directly under the dog's trunk.

Williams et al. demonstrated that the greatest increases in power during acceleration of Greyhounds occurred at the coxofemoral and tarsal joints (10). There cannot be power driving movement without stability. The pelvic limb needs to drive acceleration in the sagittal plane. Any lateral movement dissipates this power. Biomechanically, there is an inverse relationship between rear angulation and stability. In the moving dog there is a need for balance between sufficient pelvic limb angulation to provide for power for acceleration and continued movement, but also sufficient stability to apply that power effectively against the ground. This balance is thought to be achieved through moderate pelvic limb angulation as demonstrated in Figure 12.

There is strong evidence of functional trade-offs in comparing the limb muscles of dogs that have been selectively bred for running vs. fighting (11). Dogs such as Greyhounds that were bred for running have substantially less musculature in the distal limbs so that there is less weight distally and thus reduced rotational inertia of their oscillating limbs. In addition, they tend to have weaker musculature in the thoracic limbs than the pelvic limbs. The pelvic limbs are thought to have a greater role in acceleration while the thoracic limbs are more important for deceleration (24, 25).

In contrast, dogs bred for fighting, such as Pit Bulls, tend to have well-muscled distal limbs that can produce more power and sustain improved agility as well as balance and opponent manipulation (11). They also have more equal musculature in their thoracic and pelvic limbs. In these breeds thoracic limb strength is believed to be essential for rapid turning and agility. It is interesting to ponder which of these structural differences are ideal for Working Dogs, which have functions that require both acceleration and agility. As with so many other structural features, a balance between the two extremes is likely ideal.

**Figure 13:**
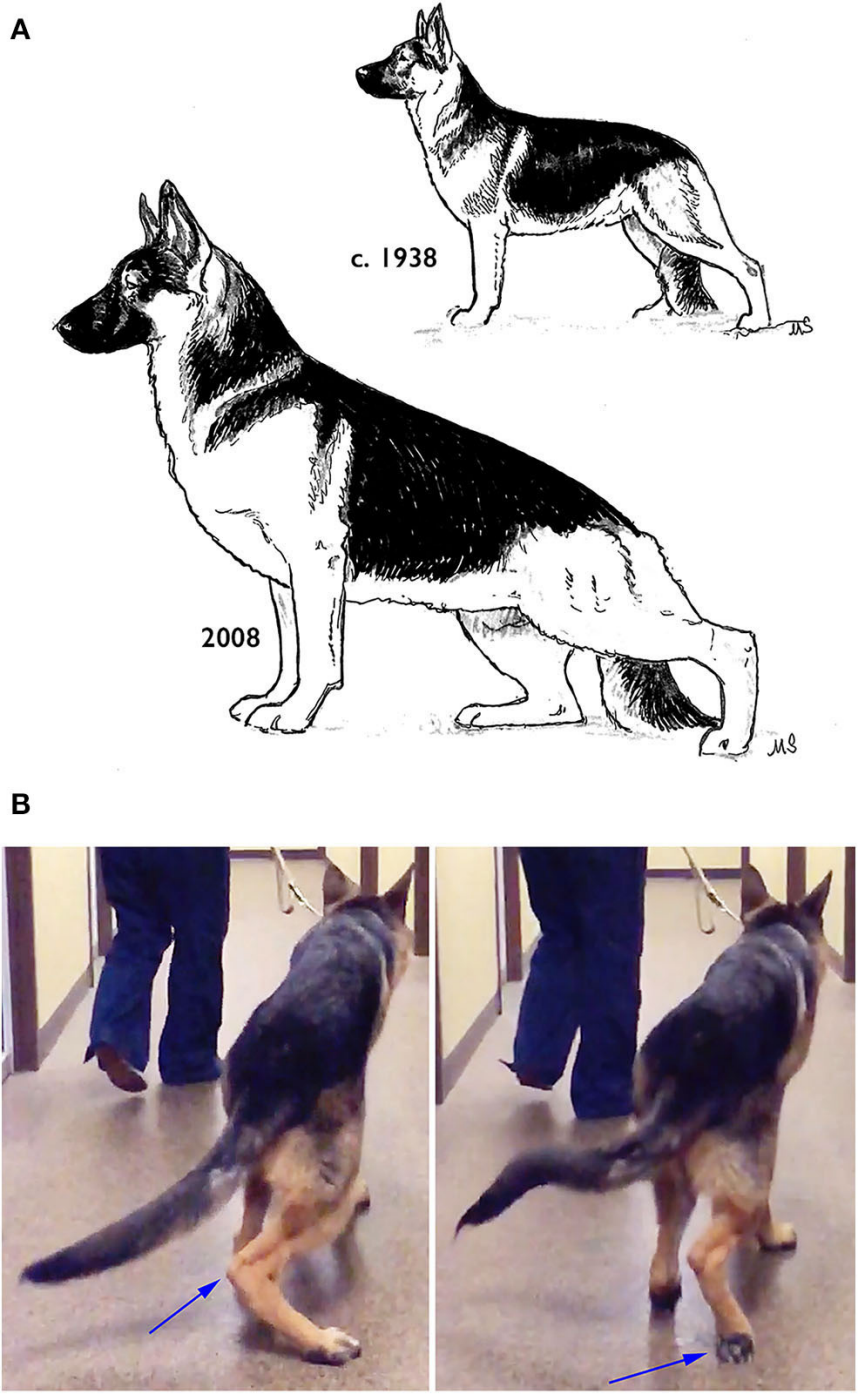
**(A)** Changes in the pelvic limb structure of the German Shepherd Dog over the last several decades. **(B)** A healthy, year-old German Shepherd Dog with extreme pelvic limb angulation showing medial displacement of the right tarsus at the end of the stance phase (left), and a knuckling of the right pelvic limb due to the inability of the left pelvic limb to support the contralateral limb during the swing phase. Illustration by M. Schlehr.

Some breeds have been selectively bred to have extreme pelvic limb angulation. One of these is the German Shepherd Dog, particularly those bred for conformation dog shows, which has shown marked increases in rear limb angulation from moderate to extremely angulated over the last several decades (Figure 13A). Many individuals of this breed have such extreme angulation that they are unable to stand in the typical stacked position but instead must stand with one pelvic limb's metatarsals perpendicular. to the ground, and with the other pelvic limb's foot placed under the body to improve stability. The result of this extreme pelvic limb angulation is that the pelvis is positioned closer to the ground, and the dog's spine is extremely sloped from cranial to caudal. This extreme pelvic limb angulation often cannot be compensated for by muscular strength, and these dogs' tarsi swing medially each time the feet are planted, thus reducing the power transmitted to the body. Often these dogs experience such instability on the standing leg that they are unable to lift the contralateral foot fully on the swing phase of the stride (Figure 13B).

To the best of our knowledge, these structural changes in the German Shepherd Dog provide no functional advantages. Any potential advantage in function appears to be offset by instability. As observed by Fischer and Lilje, “whenever selection starts, whether it is the skull or locomotion, it will affect other parts of the body” (15). The German Shepherd Dog tends to have laxity in many joints throughout the body, not just in the pelvic limb. These dogs also frequently have an increased angle of carpal extension when standing, splayed toes, etc. It is possible that this reflects an unintended selection in these dogs toward increased extensibility of all tendons and ligaments while selecting for extreme pelvic limb angulation. It is therefore perhaps not surprising that German Shepherd Dogs have a very high prevalence of hip dysplasia as compared to other large breeds with more moderate pelvic limb angulation such as Golden Retrievers, Labrador Retrievers, and Rottweilers (26). This might be one reason why many organizations are moving away from using German Shepherd Dogs as Working Dogs, or cross-breeding them with Belgian Malinois.

At the other extreme of pelvic limb angulation are breeds with very straight pelvic limb angulation. Although minimal pelvic limb angulation is more typical of breeds originally developed for guarding, some individuals of the usual Working Dog breeds can also have relatively limited pelvic limb angulation. Biomechanically, minimal pelvic limb angulation tends to increase the potential for torque along the axis of the limb and may result in increased stress on the ligaments of the stifle and tarsus. Both extremes of pelvic limb angulation should be avoided when selecting Working Dogs.

### Pelvic Limb - Rear View

**Figure 14:**
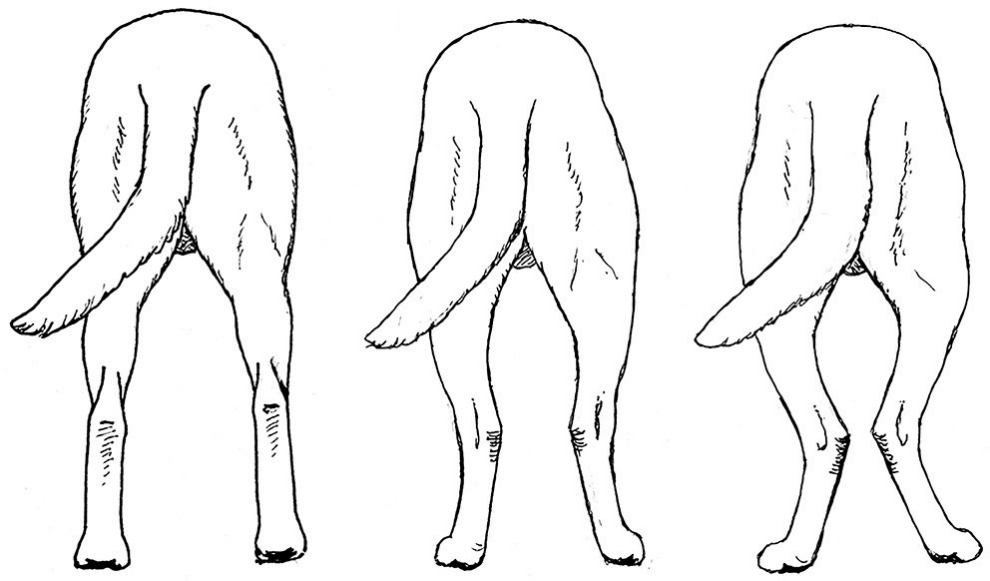
Correct pelvic limb conformation, when viewed from the rear, in the Labrador Retriever **(Left)**. The pelvic limbs of herding breeds often exhibit mild external rotation **(Middle)**, but excessive external rotation **(Right)** should be avoided. Illustration by M. Schlehr.

In many breeds, when viewed from the rear, the pelvic limbs should extend distally from the greater trochanter parallel to each other and perpendicular to the ground (Figure 14, left). Breeds such as herding dogs, whose functions require the dog to make quick turns, frequently stand with the pelvic limbs externally rotated, such that the tarsi are positioned medially relative to the stifles and feet (Figure 14, middle). This pelvic limb structure provides greater stability when the dog is required to frequently crouch, lie down and stand up. Further, it allows the toes to push off with more power when turning. This pelvic limb conformation is almost universal in German Shepherd Dogs and is very common in Belgian Malinois, both of which are herding breeds. It is less common in Labrador Retrievers, which were bred to run in straight lines to retrieve game. If this external rotation of the pelvic limbs is extreme (Figure 14, right), however, it can interfere with forward movement and should be avoided when selecting Working Dogs.

## Balanced Thoracic and Pelvic Limb Angulation

**Figure 15:**
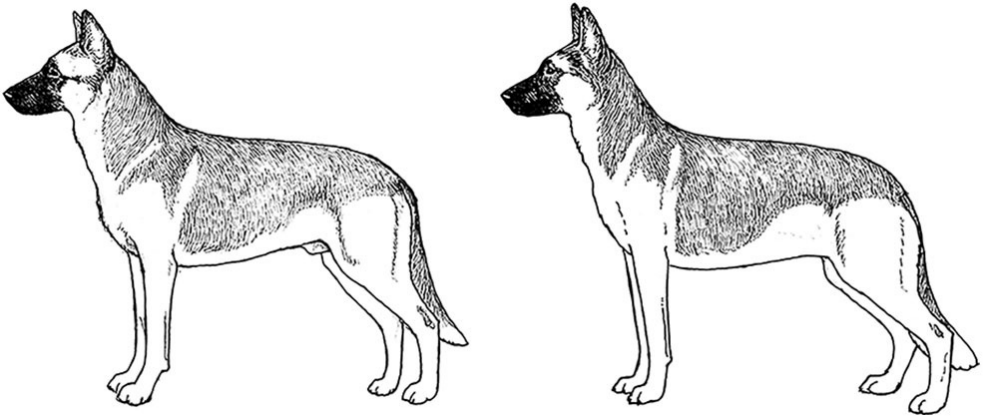
Balanced angulation in the Belgian Malinois (left). The dog on the right is imbalanced, with less thoracic limb angulation than pelvic limb angulation. Illustration by M. Schlehr.

The thoracic and pelvic limbs in a given dog should have approximately equal, or balanced, angulation. This is important for coordination of movement, particularly at the trot, when diagonally opposite thoracic and pelvic limbs strike the ground at the same time. If the thoracic limbs, for example, have less angulation than the pelvic limbs, they will have a shorter stride length and therefore a shorter cycle time than the pelvic limbs, making it difficult for diagonally opposite limbs to strike the ground at the same time. In addition, the less angulated limbs are generally less muscular than the more angulated ones. The most common form of lack of balance is when dogs have less thoracic than pelvic limb angulation (Figure 15).

For a dog to achieve the optimal thoracic or pelvic limb angulation that is consistent with its genetics, the dog's musculature must be fully developed. Further, strong muscles are required for the limbs to provide optimal power for movement. Dogs with straighter thoracic or pelvic limb angulation tend to have weaker limb musculature. This might be in part because in the standing dog, supporting the weight with bones at a more acute angle requires active muscle contraction. In a dog with less angulation a greater percentage of the dog's weight can be supported by the bones. All Working Dogs should be engaged in routine fitness programs to optimize their musculature and thus their angulation and function.

## The Head

Skull morphology is a major factor in bite force (27). Working Dogs should have large heads to provide sufficiently powerful bite muscles (predominantly the masseter and the temporalis muscles), strong jaw bones, and well-muscled necks. They also should have full dentition; a good scissors bite provides the strongest grip. Mesocephalic skulls provide the best combination of a moderate length muzzle and good teeth (28). Working Dogs also should have large, open nostrils to facilitate the passage of air when scenting.

## The Tail

The tail provides an important counterbalance for dogs when they need to turn quickly, either on land or when swimming. The tail also helps elevate the dog's rear assembly after the apex of trajectory of a jump, helping the dog land on its front feet. A Working Dog's tail should be strong and of enough length to provide sufficient counterbalance, especially for jobs that require jumping or sharp turns.

## The Coat

Working dogs need a weather-resistant coat that dries easily when wet, sheds dirt, and is easy to care for. Most Working Dog breeds have a double coat, characterized by large guard hairs that stand up from the skin supported by the undercoat, which consists of more numerous, finer hairs. Most organizations prefer to have dogs of a color that blends with the environment, so white dogs or extensive white markings are not advisable.

## Conclusion

There are many components of structure that can affect the ability of a Working Dog to achieve its optimum abilities and to have a long, injury-free career. These components are important to consider when selecting an adult dog for a career as a Working Dog. Breeders of future Working Dogs should give strong consideration to selecting for the characteristics that will allow these dogs to excel in their careers and live long and productive lives.

## Author Contributions

CZ conceived of and wrote the manuscript. MS drew all illustrations to demonstrate the specific components described in the manuscript and contributed numerous concepts and ideas to the structure and content of the manuscript. Both authors contributed to the article and approved the submitted version.

## Conflict of Interest

CZ was employed by Zink Integrative Sports Medicine. The remaining author declares that the research was conducted in the absence of any commercial or financial relationships that could be construed as a potential conflict of interest.
